# Green tea influence on iron overload in thalassemia intermedia patients: a randomized controlled trial

**DOI:** 10.12688/f1000research.25553.1

**Published:** 2020-09-16

**Authors:** Hayder Al-Momen, Hussein Khudhair Hussein, Zaid Al-Attar, Mohammed Jalal Hussein

**Affiliations:** 1 Department of Pediatrics, Al-Kindy College of Medicine, University of Baghdad, Baghdad, 00964, Iraq; 2 Department of Pharmacology, Al-Kindy College of Medicine, University of Baghdad, Baghdad, 00964, Iraq

**Keywords:** Deferasirox, Liver iron concentration, Serum ferritin, Thalassemia.

## Abstract

**Background:** Although iron chelation therapies have been available for many years for thalassemia intermedia patients, iron accumulation remains the major cause of death. Therefore, the need for additional chelation options is in demand. This randomized controlled study aimed to understand the effects of green tea on iron balance in thalassemia intermedia patients.

**Methods:** Using a random selection method, 141 thalassemia intermedia patients were initially screened for inclusion in this trial; only 68 patients included after applying exclusion criteria. Two equal groups were generated (n=34/group): green tea (three cups/day after meals) + usual treatment (deferasirox iron chelator and on demand blood transfusion); and control (only usual treatment). The study lasted for a period of 12 months. Patients failing to comply to the trial methodology were excluded, leaving a final total of 29 patients in the green tea group and 28 patients in the control group. Liver iron concentration, and serum ferritin were assessed at baseline and 12 months, while hemoglobin levels were assessed monthly.

**Results:** At baseline, both groups were matched regarding general demographics. At 12 months, the net drop of liver iron concentration in the green tea group (7.3 mg Fe/g dry weight) was significantly higher than the control group (4.6 mg Fe/g dry weight) (p<0.05). This was also seen with serum ferritin; net reduction in green tea and control groups were 1289 ng/ml and 871 ng/ml, respectively (p<0.05). Hemoglobin levels were slightly higher in the green tea group compared with the control group, but this was not significant.

**Conclusions:** Regular green tea consumption had a significant capability to improve iron deposition in thalassemia intermedia patients who already undergo deferesirox iron chelation therapy.

**Trial registration:** UMIN-CTR Clinical Trials Registry, UMIN000040841 (retrospectively registered June 21, 2020).

## Introduction

Thalassemia intermedia (TI) is part of the thalassemia syndromes, which are the commonest genetic disorders worldwide, and is a member of the non-transfusion-dependent thalassemia (NTDT) group. By definition, β-thalassemia intermedia is a hemoglobin disorder characterized by a reduction in the expression of the β-globin gene ^1, 2^. Iron overload in TI has more than one cause, the most important is increased gut absorption. In addition, ineffective erythropoiesis and hemolysis may increase the need for blood transfusions with the eventual result of iron overload ^3, 4^. High morbidity rates usually accompany iron accumulation in NTDT and TI and has a significant impact on free radicals and oxidative stress tissue damage. Iron chelators are the best solution to alleviate these pathologies ^5, 6^.

Deferasirox is the main oral iron chelation therapy according to international guidelines, but it is not free from side effects, leading to issues surrounding compliance, resulting in excessive body iron. Accordingly, recent research aims to assist, improve, or even substitute the use of iron chelating drugs using certain plants ^7, 8^. The common tea plant *Camellia sinensis* has been utilized globally as a tea drink in various subtypes based on the manufacturing process: black (fermented), green (non-fermented), and oolong (semi-fermented). Tea belongs to the group of common beverages worldwide and it is considered as the number one beverage (after water) in Iraq and some other parts of the world ^9, 10^.

Regarding green tea (GT), polyphenols are the main ingredient, representing 24–36% of dry weight. Catechins are the mainstay of these polyphenol compounds, which have inhibitory effects on iron (heme and non-heme) absorption across gut cells. This may lead to extraordinary negative iron balance in humans who consume GT as part of their daily diet ^11, 12^.

Consequently, this randomized controlled trial aimed to assess the iron accumulation levels in TI patients treated with deferasirox with additional GT intake using serum ferritin (SF) and liver iron concentration (LIC). Also, hemoglobin (Hb) levels were assessed regularly.

## Methods

### Trial design

This is a randomized controlled (parallel-group; 1:1) study performed prospectively at the Baghdad Hereditary Anemia Center at Ibn Al-Baladi Hospital in Baghdad, Iraq, which is the largest thalassemia center in the country. The study was conducted over a period from 1 ^st^ March, 2019 until 29 ^th^ February, 2020.

### Patients

All β- thalassemia intermedia patients aged ≥10 years old who were regularly managed by the Baghdad Hereditary Anemia Center were entered in a baseline screen. No sample size was calculated as all patients were considered.

Patients with chronic illnesses such as diabetes mellitus, heart failure, asthma, renal failure, liver disease, and any hematological problem other than thalassemia, were excluded from the study. Patients that failed to have full compliance with blood transfusion and iron chelation therapy according to physicians’ instructions in the last three months before baseline recruitment were also excluded.

At the initial screening, 141 patients were eligible. In total, 73 patients were excluded as they did not meet the inclusion criteria, leaving 68 patients who were randomly divided into two groups with matched age, sex, and BMI. Patients were asked to take part in the study during routine check-up appointments.

### Outcomes

Iron overload status through measurements of SF and LIC at baseline and end of the study period, in addition to regular monthly assessment of Hb levels.

### Interventions

The patients were randomly divided into two groups (n=34/group): GT group and control group.

*GT groups:* Patients in the GT group were instructed to drink a cup of GT after meals (within an hour) three times daily. Patients were given GT tea bags from Lipton™ containing 2 grams of GT ( *Camellia sinensis*), which was collected from Kenya and Indonesia and made in January 2019, in Jebel Ali, United Arab Emirates by Unilever Gulf FZE (P.O. Box 17055, Dubai). Patients were instructed to infuse the tea bag in a hot cup of water (150°C) for 2–3 minutes, according to the manufacturer’s instructions. Patients were undergoing deferasirox treatment and blood transfusion (when needed as decided by the attending physician) as their usual treatment.

*Control group:* Patients in the control group were instructed drink one cup or more of either warm or cold drinking water after each meal, and to avoid any beverage other than water for three hours after each meal daily. Patients were undergoing deferasirox treatment and blood transfusion (when needed as decided by the attending physician) as their usual treatment.

Patients of both groups were asked to report their daily meals through a phone call (twice a week) to ensure low-iron food consumption, which might be considered as a confounding factor. Follow-up visits were performed at least every month with the patients attending physician.

During the study interval (12 months), participants failing to follow these instructions for three days a month (a total of nine drinking events during 30 days, which represents 10%) were excluded from analysis.

### Randomization

Simple randomization was performed to allocate participants into GT and control groups in an equal pattern with matched age, sex, and BMI. Researchers have given members of each group a unique sequential number which was applied to all laboratory blood samples and MRI results. The researchers have enrolled and assigned participants to GT and control groups. The attending physicians and staff of the laboratory and MRI units were blinded to the study groups.

### Data collection

Full medical history and examination with the decision for blood transfusion requirement and iron chelation therapy (deferasirox 20–40 mg/kg/day) were made by the attending physician during each follow-up visit (at least once a month) during the study period. Body mass index (BMI (kg/m2)) was calculated at the start of the study for all patients. Also, packed red blood cells (RBC) transfusion volume during the previous year, calculated just before the start of study period, was estimated for all participants.

The following measurements were taken at baseline and 12 months for all patients:

- Serum ferritin (SF) readings (ng/ml), determined using a MINI VIDAS analyzer (bioMerieux S.A., Lyon, France) through an electrofluorescence assay method;
- Liver iron concentration (LIC) values (mg Fe/dry gram weight), determined using R2 magnetic resonance imaging (MRI; Siemens MRI machine with 3 Tesla and Medis suite software);
- Hemoglobin levels (g/dl) (documented monthly), determined using microprocessor hemoglobin meter (model number LE182, and manufactured by GPC Medical Ltd.)

### Statistical analysis

Data are expressed as mean±standard deviation (SD) unless otherwise indicated. Shapiro-Wilk test was used for normal distribution. Pearson's chi-square test (for discrete or nominal variables) or Student t-tests (for continuous variables) were used to compare the characteristics between GT and control groups. When p < 0.05, results were assumed to be significant. SPSS 22.0 for Windows (IBM Corp., NY, USA) was utilized in the analysis.

### Ethical considerations

Ethical approval was obtained from Ethical and Scientific Committee of Al-Kindy College of Medicine at University of Baghdad (No.: 367). Baghdad Hereditary Anemia Center is affiliated with Al-Kindy College of Medicine at University of Baghdad. Written informed consent for participation was taken from all participants or their caregivers/legal guardians for those aged <18 years. All work was done according to the most recent version of the Declaration of Helsinki. The trial was registered retrospectively on UMIN-CTR Clinical Trials Registry UMIN000040841 (June 21, 2020) according to the University of Baghdad recommendations.

## Results

**Figure 1.**
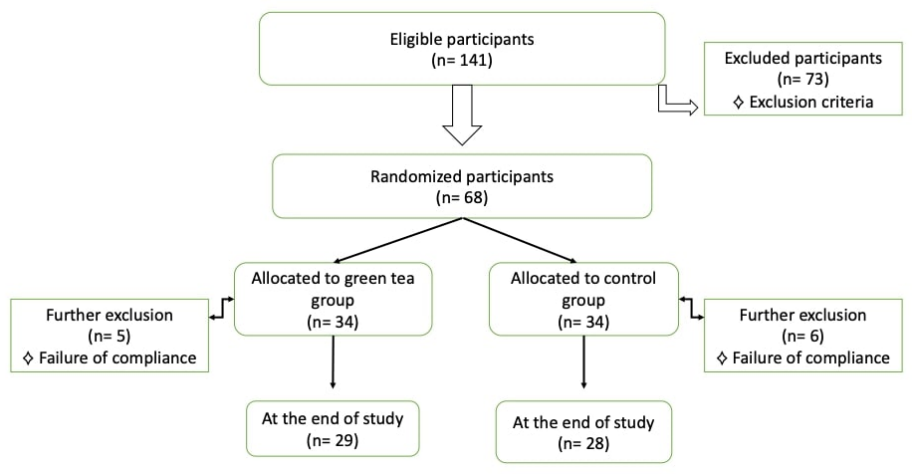
Flowchart of participants.

Due to non-compliance, the GT group had 29 patients (five patients excluded) and the control group had 28 patients (six patients excluded) at the end of the study ( Figure 1).

The two groups had comparable (non-significant) general demographic data at baseline ( Table 1). Iron overload (measured by SF or LIC) and volume of packed red blood cells (RBC) transfusion during the previous year calculated just before the start of study period showed no significant differences between groups. Deferasirox mean doses for the GT (36.89±7.24 mg/kg/day) and control (37.21±1.13 mg/kg/day) groups were high and near to the maximum approved doses, but were not significantly different between the groups.

**Table 1.** Participant characteristics at baseline.

Variable	Green tea group (n=29)	Control group (n=28)	P value
Age, years (mean±SD)	13.62±4.27	13.47±2.58	0.81 ^a^
Male:female	15:14 (51.72%:48.28%)	14:14 (50%)	
Body mass index, kg/m ^2^ (mean±SD)	18.45±4.91	19.11±3.40	0.63 ^a^
Deferasirox dose, mg/kg/day (mean±SD)	36.89±7.24	37.21±1.13	0.75 ^a^
Pre-transfusion, hemoglobin g/dl (mean±SD)	8.21±3.36	8.16±4.15	0.86 ^a^
Packed red blood cell transfusion volume during the year of pre-study period, ml/kg (mean±SD)	71.84±9.97	73.42±9.53	0.41 ^a^
Splenectomized, n (%)	7 (24.14)	7 (25.07)	0.90 ^b^
Serum ferritin, ng/mL (mean±SD)	2617±549	2684±164	0.57 ^a^
Liver iron concentration, mg Fe/g dry weight (mean±SD)	19.4±2.3	19.8±1.4	0.84 ^a^

a: Student t-tests, b: Pearson's chi-square test.

**Figure 2.**
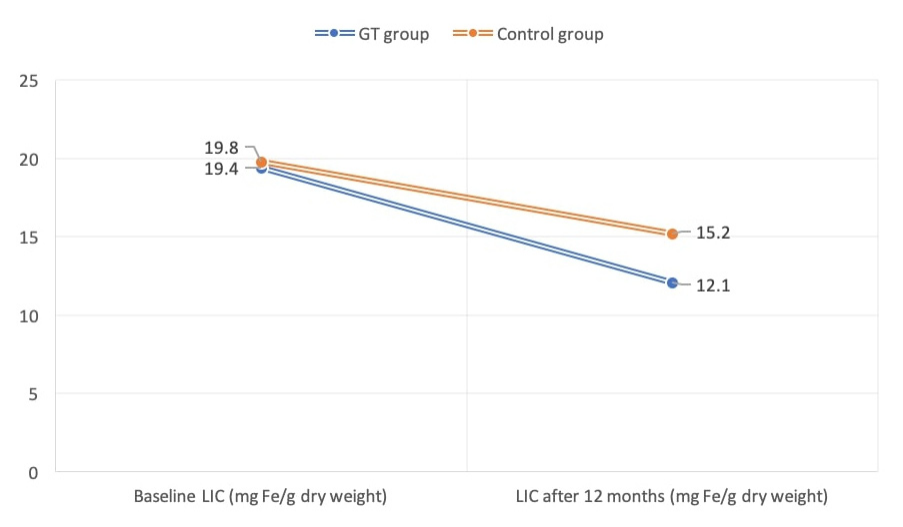
Liver iron concentration (LIC) at the start and end of study period in the green tea (GT) and control groups.

LIC was significantly reduced (P<0.05) after 12 months in both the GT group (baseline, 19.4±2.3 Fe/gram/dry weight; 12 months, 12.1±1.3 Fe/gram/dry weight), and control groups (baseline, 19.8±1.7 Fe/gram/dry weight; 12 months, 15.2±1.6 Fe/gram/dry weight) ( Figure 2). Similarly, SF levels were significantly decreased at the end of study period (P<0.05) in the GT (baseline, 2617±549 ng/ml; 12 months, 1328±127 ng/ml) and control (baseline, 2684±164 ng/ml; 12 months, 1813±235 ng/ml) groups.

Deferasirox use was comparable at the start and end of the study period for both the GT (baseline, 36.89±7.24 mg/kg/day; 12 months, 36.24±6.83 mg/kg/day) and control (baseline, 37.21±1.13 mg/kg/day; 12 months, 37.15±3.78 mg/kg/day) groups.

The mean volume of transfused packed RBC during the study period was estimated after 12 months from the baseline and revealed no statistical significance between GT (70.64±7.84 ml/kg) and control (72.14±8.35 ml/kg) patients. The net reduction of LIC (Fe/gram/dry weight) in GT patients was 7.3 (baseline, 19.4; 12 months, 12.1), while for control patients the net result was 4.6 (baseline, 19.8; 12 months, 15.2). A significant reduction (P<0.05) of LIC was found in the GT group in comparison with the control cases. The drop of serum ferritin in GT patients after 12 months [1289 ng/ml (2617-1328)] was significantly (P<0.05) higher than that of the control group [871 ng/ml (2684-1813)] ( Table 2).

**Table 2.** Relationship between specific events within the study groups.

Event	Green tea group (n=29)	Control group (n=28)	P value
Packed red blood cell transfusion volume (ml/kg) over 12 months (mean±SD)	70.64±7.84	72.14±8.35	0.37 ^a^
Net drop of mean liver iron concentration (mg Fe/g dry weight) at the end of the study	7.3	4.6	< 0.05 ^a^
Net drop of mean serum ferritin (ng/ml) at the end of the study	1289	871	< 0.05 ^a^

a: Student t-tests.

**Figure 3.**
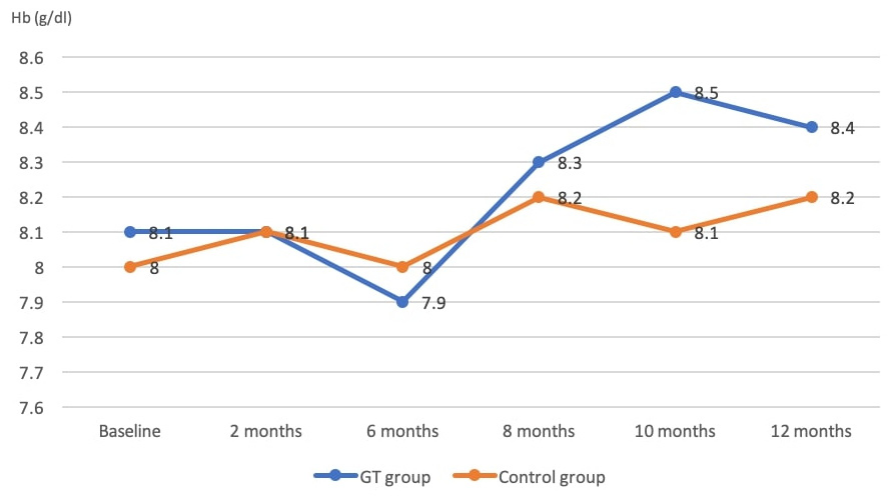
Hemoglobin (Hb) levels in the green tea (GT) and control groups throughout the study (measured monthly).

Hemoglobin readings gradually increased in both the GT and control groups over the study period. GT patients had higher Hb levels after 12 months than the control group (8.4 versus 8.2), but this was not statistically significant (P=0.37) ( Figure 3).

## Discussion

Iron overload is a major consequence of TI. Physicians have several challenges when using medications that eliminate excessive iron, including type, dose, expected adverse effects, and the best time to start an iron chelator. Therefore, the need for a naturally mediated iron chelator based on plants is increasing along with the increase of thalassemia patients around the world ^13– 15^. Green tea (GT) seems to be one of the best available options due to its natural properties to counteract iron absorption in addition to other promising advantages ^16, 17^.

There are several ways to estimate the overload of iron in thalassemia patients, such as the widely available test of SF, and the most accurate non-invasive recent method of LIC using the R2 MRI technique ^18, 19^.

Patients with TI usually have high iron content loading in their tissues as evidenced by other studies from different areas of the world ^13, 20, 21^. Our sample of patient had even higher iron content, which is in line with other reports from Middle East ^3, 14^. This might be due to compliance deterioration, background cultural beliefs, possible side effects of iron chelating drugs, and the frequent use of mimic medications in this part of the world ^22^.

GT consumption in our sample was associated with decreased ferritin levels mirroring better iron chelation. This was also noticed by other works done on both humans and animals with transfusion dependent thalassemia ^23– 25^. SF could be affected by the antioxidant properties of GT ^26, 27^. However, a recent study that added curcumin to GT for thalassemia patients discovered a slight increase of SF after two months, with the reasons for this unknown to the authors ^28^.

A more sophisticated method of iron accumulation assessment in TI is LIC, and in this study, we found a significant drop in the GT group compared to the control group. This has only been previously reported in animal studies, and an online search conducted by the present authors failed to reveal human trials ^15^. It should be noted that iron deficiency has been documented in healthy individuals consuming large amounts of GT ^29^.

Surprisingly, in the GT group, TI patients developed slightly higher hemoglobin readings than controls. Again, no previously published data was found to verify this, but it might be related to better iron removal and effects of antioxidant characteristics of GT, which could be associated with improved hematopoiesis and less red blood cells hemolysis. However, GT is historically associated with anemia in a healthy population, especially in high doses ^30^.

Although this trial was conducted in the largest thalassemia center within Iraq country borders, its small sample size could be considered as a limitation. We recommend other interested scientists throughout the world to involve more patients with more variables and risk factors to deeply investigate the effects of GT in TI and discover more plant-based iron chelation therapies with better effects.

## Conclusion

Thalassemia intermedia patients having usual medical care, including blood transfusion and deferasirox oral iron chelation therapy, have significantly superior levels of iron chelation and slightly better hemoglobin levels when consuming GT regularly compared with a control group.

## Data availability

### Underlying data

Mendeley Data: Green tea influence on iron overload in thalassemia intermedia, http://dx.doi.org/10.17632/p7558gcrmw.2 ^31^.

Data are available under the terms of the Creative Commons Attribution 4.0 International license (CC-BY 4.0).

### Reporting guidelines

Mendeley Data: CONSORT checklist for ‘Green tea influence on iron overload in thalassemia intermedia patients: a randomized controlled trial’, http://dx.doi.org/10.17632/9c5bx4p65c.1 ^32^.
