# Supplementary material for: Green tea influence on iron overload in thalassemia intermedia patients: a randomized controlled trial
Source: F1000Res. 2020 Sep 16;9:1136. [Version 1] doi: 10.12688/f1000research.25553.1 (PMC7520712; doi:10.12688/f1000research.25553.1)
Supplement: Peer review history

# Peer review: Reviewer response for version 1

**DOI:** 10.5256/f1000research.28201.r71683

**Published:** 2020-09-25

**Authors:** Hassan Abolghasemi

**Affiliations:** 1 Faculty of Medicine, Baqiyatallah University of Medical Sciences, Tehran, Iran

In this article the impact of green tea on reduction of liver iron concentration have been studied in a randomized clinical trials by authors. They showed liver iron was decreased in the case groups compared with control group significantly. The level of hemoglobin was not significantly changed at the end of trial.

 The trial was done appropriately.

 The only problem with the study was the level of ferritin at the start of trial which was higher than usual for thalassemia intermedia and the dose of Deferasirox. It seems that some of the patients were transfusion dependent.

Is the work clearly and accurately presented and does it cite the current literature?

Yes

If applicable, is the statistical analysis and its interpretation appropriate?

Yes

Are all the source data underlying the results available to ensure full reproducibility?

Yes

Is the study design appropriate and is the work technically sound?

Yes

Are the conclusions drawn adequately supported by the results?

Yes

Are sufficient details of methods and analysis provided to allow replication by others?

Yes

Reviewer Expertise:

pediatric hematology

I confirm that I have read this submission and believe that I have an appropriate level of expertise to confirm that it is of an acceptable scientific standard.

# Peer review: Reviewer response for version 1

**DOI:** 10.5256/f1000research.28201.r71684

**Published:** 2020-09-24

**Authors:** Alaa Alwan

**Affiliations:** 1 Department of Clinical Hematology, National Center of Hematology, Mustansiriyah University, Baghdad, Iraq

- It is interested subject which has covered important issue in beta thalassemia.
- The introduction is well covered with recent references.
- Materials and method done appropriately especially with use of statistical methods and randomization.
- Regarding the results, they are clear and in my opinion it is obvious.
- The discussion part is well written and it is good to see comparison with other studies.

Is the work clearly and accurately presented and does it cite the current literature?

Yes

If applicable, is the statistical analysis and its interpretation appropriate?

Yes

Are all the source data underlying the results available to ensure full reproducibility?

Yes

Is the study design appropriate and is the work technically sound?

Yes

Are the conclusions drawn adequately supported by the results?

Yes

Are sufficient details of methods and analysis provided to allow replication by others?

Yes

Reviewer Expertise:

benign and malignant hematology

I confirm that I have read this submission and believe that I have an appropriate level of expertise to confirm that it is of an acceptable scientific standard.
